# Understanding parents' use of a knowledge translation tool to manage children's vaccination pain

**DOI:** 10.1097/PR9.0000000000000907

**Published:** 2021-03-11

**Authors:** Nicole E. MacKenzie, Perri R. Tutelman, Christine T. Chambers, Jennifer A. Parker, Noni E. MacDonald, C. Meghan McMurtry, Pierre Pluye, Vera Granikov, Anna Taddio, Melanie Barwick, Kathryn A. Birnie, Katelynn E. Boerner

**Affiliations:** a Department of Psychology and Neuroscience, Dalhousie University, Halifax, NS, Canada; b Centre for Pediatric Pain Research, IWK Health Centre, Halifax, NS, Canada; c Department of Pediatrics, Dalhousie University, Halifax, NS, Canada; d Department of Psychology, University of Guelph, Guelph, Ontario, Canada, and Pediatric Chronic Pain Program, McMaster Children’s Hospital, Hamilton, Ontario, Canada,; e Department of Family Medicine, McGill University, Montreal, QC, Canada; f Leslie Dan Faculty of Pharmacy, University of Toronto, Toronto, ON, Canada; g Child Health Evaluative Sciences, Research Institute, The Hospital for Sick Children, Department of Psychiatry, Faculty of Medicine, University of Toronto, and Dalla Lana School of Public Health, University of Toronto, Toronto, Ontario, Canada; h Department of Anesthesiology, Perioperative and Pain Medicine, University of Calgary, Calgary, AB, Canada; i Department of Psychiatry, BC Children's Hospital, University of British Columbia, Vancouver, BC, Canada

**Keywords:** Vaccination, Knowledge translation, Pain management, Pediatric pain, Evidence-based practice, Vaccine hesitancy

## Abstract

**Introduction:**

Although several evidence-based strategies for managing children's vaccination pain exist, many parents report being unaware of them. Knowledge translation (KT) tools present evidence-based information in plain language.

**Objectives:**

This two-phase study assessed parents/caregivers' uptake of evidence-based pain management strategies via a KT tool and considered factors related to parents' planned, actual, and future use of these strategies.

**Methods:**

In phase 1, parents were exposed to an online KT tool on physical, psychological, and pharmacological vaccination pain management strategies, and their impressions were assessed by questionnaires including the Information Assessment Method for Parents. In phase 2, after vaccination, parents completed a follow-up survey on their uptake and experiences using the information.

**Results:**

A total of 312 participants reported their plans for KT tool use. Parents who found the KT tool relevant were more likely to plan to use it at their child's upcoming vaccination. A total of 128 parents (93% mothers) completed both surveys. Nearly all parents who planned to use the information did so during their child's subsequent vaccination (90%). When the KT tool was relevant to their needs, parents were more likely to use the information during their child's vaccination. Parents who felt confident using the tool were significantly more likely to report plans for future tool use.

**Discussion:**

This study demonstrates the effectiveness of a KT tool that was relevant to parents' needs and built confidence to increase parent-reported uptake of evidence-based strategies. Proper pain management could positively impact parents' uptake of vaccinations for children.

## Abstract 2

Supplemental Digital Content is Available in the Text.

The relevance of KT tools, and parents' confidence in using them, was related to uptake of vaccination pain management strategies for children.

## 1. Introduction

Pain associated with vaccination has been identified as a contributor to parental/caregiver (parents and caregivers refer to those who are responsible for the health and well-being of the child and will be referred to as “parents” throughout this manuscript) vaccine hesitancy (ie, delay or refusal of vaccination despite access and availability) and nonacceptance of vaccines as recommended by the relevant schedule.^43^ The World Health Organization has identified vaccine hesitancy as one of the 10 threats to global health.^50^ Concerns regarding vaccination pain are not only problematic for children's health, but also have implications for acceptance of vaccinations into adulthood.^16^ Clinical practice guidelines have identified and described a range of evidence-based vaccination pain management strategies including ones that target children.^45^ Given that parents are typically present when their children are being immunized in traditional medical settings, they are uniquely positioned to manage their children's vaccination pain.^11^ Despite this, parents report poor knowledge of pain management strategies for children's vaccinations,^41^ Furthermore, although parent-directed resources regarding vaccination are available, they are not often paired with pain management information.^44^ Thus, there is a disparity in access to and use of child-appropriate, evidence-based vaccination pain management strategies for children.^24^

This gap between evidence-based practices and their access, knowledge, and use by parents can be addressed by dissemination through knowledge translation (KT), defined as “…a dynamic and iterative process that includes synthesis, dissemination, exchange, and ethically sound application of knowledge to improve the health of Canadians, provide more effective health services and products, and strengthen the healthcare system.”^8^ Research has shown that patient-directed KT interventions (ie, those that actively engage patients/caregivers to enhance knowledge, service use, and health behaviour and outcomes) show moderate-to-large impacts on care delivery and uptake of evidence-based practices.^12,38^

Knowledge translation tools serve as a bridge for communication with knowledge users, yet it is critical to understand if and how the tools are used. In a study where evidence-based pain management information was passively shared with parents (ie, included in a postnatal hospital discharge information package), there was a 10% increase in evidence use among parents who reviewed the tool.^44^ In clinical settings, however, parents may have expectations that integration of evidence into practice is the healthcare provider's (HCP) role, given their reliance on HCPs as a source of information for supporting children through vaccinations.^23^ Uptake and application of evidence, such as clinical practice guidelines, is shown to increase in clinical environments when HCPs provide guidance on how to implement the intervention based on evidence for what makes the intervention effective.^1,35^ This may also be relevant to understanding parents' uptake of KT interventions and tools and their needs in the clinical environment when it comes to their use. Although there has been an increasing number of parent-directed KT tools, there is little understanding of what promotes their uptake of evidence.^13^

In an effort to close the gap between evidence and uptake, a clinical practice guideline on best practices for vaccination pain management was published,^45^ from which a two-page KT tool for parents was developed (see Appendix for the KT tool, available at http://links.lww.com/PR9/A97). The KT tool provided a plain language summary of evidence-based practices for parents regarding pharmacological, psychological, and physical strategies for managing children's vaccination pain. This freely available tool (https://issuu.com/parentscanada1/docs/parentscanada-ad-feature-needles-do?e=32636145/58777569) was circulated nationally in partnership with *ParentsCanada* magazine, a parenting magazine. Although the KT tool reached over 80,000 parents,^4^ the impact of this tool on pain management strategy uptake for children's vaccination pain is unknown.

Methods of assessing whether KT tools achieve their stated objectives are important to understanding what works, why, and for whom. This can be understood through psychological and cognitive components of information use, as some theoretical frameworks would indicate,^14,31^ as well as parents' attitudes toward vaccines,^6^ which may influence parents' interest in, and uptake of, evidence-based practices for vaccination pain management.

A related concept is parents' confidence in their ability to use evidence. Parental confidence in being able to carry out a behaviour change intervention has been related to actual use of the given strategies to change behaviour and increased knowledge about a given behaviour.^18,20^ Thus, confidence may be influenced by education on, and experience implementing, a given evidence-based tool. This, however, has not explicitly been studied in the KT or vaccination literature.

By evaluating these concepts within the KT tool, it creates the opportunity to identify specific variables that promote parents' uptake of evidence-based practices to manage their children's vaccination pain. Thus, the objectives of this study were to understand what variables relate to participant's plans to use the KT tool, whether participants' plans to use the tool related to actual strategy use, and whether confidence in strategy use related to plans to continue using the tool at future vaccination appointments. It was hypothesized that factors related to the Information Assessment Method (IAM) variables and perceived confidence in using the tool use would predict plans to use the tool, and that plans to use the tool would predict actual use. It was also hypothesized that participants' confidence in using the tool during their child's vaccination would predict plans to continue using the tool at future vaccinations.

## 2. Methods

### 2.1. Participants

Parents of children aged 0 to 17 years, who were English-speaking and had either previously vaccinated their children or had plans to vaccinate in the future, were recruited to participate in this two-phase, online study. Participants who had not previously vaccinated their children or did not have plans to in the future were excluded. Participants were recruited through convenience sampling on social media, e-newsletters, and website posts, with recruitment open to international participants as well.

### 2.2. Procedure

The study was approved by the IWK Health Centre Research Ethics Board, and participants provided informed consent online in each study phase. The study consisted of 2 phases.

#### 2.2.1. Phase 1

In Fall 2018, participants were recruited to participate in the phase 1 survey, before a child's upcoming vaccination appointment. Participants viewed the aforementioned KT tool on children's vaccination pain management strategies online, embedded within the survey. The KT tool provided physical (eg, positioning), psychological (eg, distraction), and pharmacological (eg, topical anesthetic) strategies for pain management for infants, school-age children, and adolescents. After review of the KT tool, participants subsequently completed a brief online survey immediately after they finished viewing the tool. Online recruitment was open for one month.

#### 2.2.2. Phase 2

Phase 2 was initiated approximately 6 months later in Spring 2019, after children's recent vaccinations, and participants from phase 1 were recontacted to complete a follow-up online survey on their child's vaccination status and KT tool use.

In line with best practices in patient-oriented research,^10^ both surveys were piloted with patient partners (where “patient” refers to individuals who have experience with a health issue, including family members)^9^ to ensure clarity and relevance of questions and information. These parents included 2 mothers and one father whose children experienced pain related to a health condition. Participants who completed each respective survey were offered the opportunity to enter into a random draw for a $100 online gift card.

### 2.3. Measures

#### 2.3.1. Phase 1 survey

This survey consisted of 3 primary components. The first component was the *IAM-Parents* questionnaire, a 6-item measure that assesses how parents interact with health resources. The IAM is based on a theoretical framework that can be used to understand the effect of engagement with the KT tool on parents' uptake of evidence-based strategies for their children's vaccination pain and other health-related decision making.^14,31^ It evaluates these concepts through 4 domains: plans to use the information, perceived relevance of parenting resources, cognitive effects of the information, and anticipated benefits of resource use.^32^ This questionnaire, which has been shown to have very good ecological, content, and construct validity, as validated with parents of young children,^7^ adapts the IAM framework and tailors the questions specifically to parent-directed resources. The IAM outcomes measured through the IAM-Parents are the situational relevance of the information, cognitive impact of information (ie, whether the information aligns with an individual's attitudes, provides new information, or provides reassurance), intention to use information, and anticipated benefits (eg, patient satisfaction and reduction of anxiety).^31^ Together, these variables create a composite of factors that contribute to the impact that health information has on an individual and how they interact with such materials.

The second component was the *Parent Attitudes toward Childhood Vaccinations* (PACV) questionnaire, a 15-item measure that assesses parents' beliefs and attitudes about childhood vaccinations to gauge vaccine hesitancy.^26,27^ The PACV is shown to have very good construct validity as well as internal consistency.^25,28^ This questionnaire derives a composite score ranging from 0 to 100, where scores of 50 or greater indicate vaccine hesitancy.

The third survey component included questions created for use in this study and asked participants about their perceived confidence in potentially using the tool to manage their child's pain during a vaccination in general. Confidence was rated on a 5-point Likert scale, ranging from “very confident” to “not at all confident.” This component also inquired as to when participants planned to next vaccinate their child or children. Finally, demographic characteristics were collected.

#### 2.3.2. Phase 2 survey

Eligibility for phase 2 required participation in phase 1, and also required parents to have had their child or children vaccinated since completing phase 1. Participants who had consented to further research in phase 1 were contacted via email to participate in phase 2 data collection. The phase 2 survey, created for use in this study, consisted of a series of questions asking participants about whether their child or children had recently been vaccinated, and if so, whether they used strategies from the KT tool at the appointment. To address these questions, and others around participants' experiences using the KT tool, the survey included an adapted version of the IAM-Parents questionnaire. The adapted IAM-Parents was modified to ask the same items as the original version used in phase 1, but in reference to a recent vaccination. Finally, participants reported on their confidence in using the pain management strategies at a child's recent vaccination and were also asked to indicate whether they would plan to use the tool again in the future.

### 2.4. Sample size and statistical analyses

Sample size was calculated and determined to require at least 80 participants (power [1 − β] at 0.95, α = 0.05) for both study phases. Data analysis was performed using Statistical Package for the Social Sciences (SPSS) Version 23 (IBM Corp., Released 2015, IBM SPSS Statistics for Windows, Version 23.0. Armonk, NY). Descriptive statistics examined participant demographics, vaccine hesitancy, IAM variables and confidence related to use of the KT tool, vaccination rates, and strategy use. Wilcoxon signed-rank tests and chi-square analyses were used to examine differences between IAM variables and confidence between the 2 study phases. Binary logistic regressions (forward selection) were used to predict the odds of the IAM variables influencing plans to use the tool at an upcoming vaccination, tool use at phase 2, and the likelihood of using the tool again in the future.

## 3. Results

### 3.1. Participants

A total of 312 participants completed the phase 1 survey. A final sample of 128 participants participated in the follow-up phase 2 survey. Participants were predominantly mothers, White/Caucasian, aged between 30 and 49 years, and had a university education (Table 1). The predominance of mothers in the sample is representative of actual practices, where mothers typically accompany their children to medical appointments.^15^ Over 98% did not endorse vaccine hesitancy as assessed by the PACV (Table 1).

**Table 1:** Demographics.

	Prevaccination (phase 1), % (n)	Postvaccination (phase 2), % (n)
Parent		
Mother	92.9 (290)	81.0 (119)
Father	5.4 (17)	5.4 (8)
Other	0.6 (2)	0.0 (0)
Age		
Younger than 20	0.3 (1)	0.0 (0)
20–29	4.5 (14)	5.4 (8)
30–39	57.4 (179)	60.5 (89)
40–49	32.4 (101)	19.7 (29)
50–59	3.5 (11)	1.4 (2)
Prefer not to answer	0.6 (2)	0.0 (0)
Level of education		
High school graduate	2.2 (7)	1.4 (2)
College graduate	11.9 (37)	8.8 (13)
Some university	4.8 (15)	2.0 (3)
University graduate	31.1 (97)	30.6 (45)
Graduate degree/professional training	48.4 (151)	44.2 (65)
Prefer not to answer	1.3 (4)	0.0 (0)
Current marital status		
Married/common-law	91.0 (284)	79.6 (117)
Divorced/separated	3.5 (11)	4.1 (6)
Widowed	0.3 (1)	0.7 (1)
Never married	2.6 (8)	2.0 (3)
Prefer not to answer	0.7 (1)	0.0 (0)
Ethnicity		
Indigenous	1.0 (3)	0.7 (1)
Arab/West Asian	5.8 (18)	3.4 (5)
Black	2.2 (7)	3.4 (5)
East Asian	2.9 (9)	1.2 (2)
South Asian	2.2 (7)	0.7 (1)
Latin American	1.9 (6)	3.4 (5)
White/Caucasian	83.7 (261)	75.5 (111)
Other	6.1 (19)	1.4 (2)
Prefer not to answer	0.6 (2)	0 (0)
Vaccine hesitancy		
Vaccine hesitant	1.9 (6)	1.6 (2)
Not vaccine hesitant	98.1 (303)	98.4 (126)

Phase 1: *N* = 312; Phase 2: *N* = 128; Vaccine hesitancy assessed using PACV questionnaire. Categories with no responses omitted from table.

PACV, Parent Attitudes toward Childhood Vaccination.

### 3.2. Phase 1

#### 3.2.1. Information Assessment Method domains and confidence

The majority of participants found the KT tool to be very relevant (66%, n = 207/312) and reported understanding the content very well (81%, n = 253/312). Most participants anticipated benefits from using the KT tool (86%, n = 276/312) and intended to use a strategy presented (94%, n = 293/312). The KT tool had a positive cognitive impact on the majority of participants (94%, n = 293/312) and either felt confident (44%, n = 136/312) or very confident (42%, n = 132/312) in their ability to use the strategies to help their child cope with future vaccination pain.

#### 3.2.2. Predicted probability of plans to use information

A series of chi-squared models were conducted to examine the IAM variables and confidence in relation to participant plans to use a strategy and the most parsimonious was selected to run the binary logistic regression. This model included confidence and relevance as the independent variables (Table 2). Confidence was not found to have a significant effect on plans to use a strategy at a subsequent vaccination (β = 0.20, *P* = 0.97); however, relevance did predict a greater probability of planning to use a strategy (β = 1.96, *P* < 0.01). Participants were 7.12 times more likely to plan to use a strategy if they found the information relevant to their context than if they did not.

**Table 2:** Coefficients of the model predicting whether participants would plan to use a strategy.

Variable	*b*	95% confidence intervals for odds ratio		
		Lower	Odds	Upper
Included				
Constant	−3.43			
Confidence	0.20	0.46	1.02	2.27
Relevance	1.96*	2.64	7.12	19.23

*R*^2^ = 2.69 (Hosmer and Lemeshow), 0.06 (Cox and Snell), 0.20 (Nagelkerke). Model χ^2^ (2) = 18.05, *p* < 0.001.

* *p* < 0.01.

### 3.3. Phase 2

In total, 102 participants (of the complete sample of 128) reported vaccinating their children at phase 2. A total of 26 (of the total 128) participants were not eligible to participate because they did not vaccinate their children within the time between the study phases and thus did not complete the phase 2 survey. Common reasons for not vaccinating children included a vaccination was not due (n = 18/26), time restraints (n = 3/26), and participants choosing not to take their children for influenza vaccinations (n = 2/26). In terms of demographics, these participants did not differ significantly from phase 1. Children were administered routine vaccinations (69%, n = 71/102) and influenza vaccinations (74%, n = 76/102), sometimes concurrently. Overall comparisons were made across study phases.

#### 3.3.1. Strategy use

Plans to use a strategy at a subsequent vaccination appointment in phase 1 were compared with actual use of a strategy at a recent vaccination. A total of 97% (n = 99/102) of respondents planned to use a strategy, whereas 87% (n = 89/102) actually did so at the time of vaccination; however, this difference was not significant (*P* = 0.34, Fisher exact test). The primary reason for not using a strategy was that the information was not found to be relevant (30.8%, n = 4/13), followed by forgetting to use the tool (23%, n = 3/13). Distraction was the most commonly used strategy (74%, n = 66/89; Table 3).

**Table 3:** Strategies used from knowledge translation (KT) tool.

	% (n)
Distraction	74.2 (66)
Holding baby	70.8 (63)
Prompting child	50.6 (45)
Deep breathing	24.7 (22)
Breastfeeding	23.6 (21)
Numbing cream	19.1 (17)
Most painful vaccination last	16.9 (15)
Sucrose	3.4 (3)
Muscle tension	2.2 (2)

Strategy use only calculated for participants who vaccinated their children and used a strategy in phase 2 (*n* *=* 89). Participants could make multiple selections.

#### 3.3.2. Information Assessment Method domains, confidence, and future strategy use

Most participants found the KT tool to be either very relevant or relevant at the time of vaccination (Table 4). The majority of participants (75%, n = 67/102) reported understanding the information in the KT tool very well at the time of vaccination and also perceived benefits as a result of strategy use (93%, n = 83/102). Nearly all participants (99%, n = 88/102) endorsed positive cognitive impact (ie, positive impression of the information, expected improvement in child's well-being) and they felt very confident or confident in their strategy use (94%, n = 89/102). Most participants reported being very likely to use a vaccination pain management strategy again at a future vaccination (76%, n = 68/102).

**Table 4:** Information Assessment Method and other outcome variables prevaccination and postvaccination.

Outcome variable	Prevaccination (phase 1), % (*n*)	Postvaccination (phase 2), % (*n*)
IAM variable		
Relevance		
Not very relevant	0.0 (0)	0.0 (0)
Somewhat relevant	1.1 (1)	16.9 (15)
Relevant	12.4 (11)	41.6 (37)
Very relevant	86.5 (77)	41.6 (37)
Understanding		
Very poorly	0 (0)	0 (0)
Poorly	0 (0)	0 (0)
Well	12.4 (11)	24.7 (22)
Very well	87.6 (78)	75.3 (67)
Benefits		
Yes	94.4 (84)	93.3 (83)
No	2.2 (3)	5.6 (5)
Positive cognitive impact		
Yes	95.5 (85)	98.9 (88)
No	4.5 (4)	1.1 (1)
Confidence		
Not at all confident	0.0 (0)	0.0 (0)
Not very confident	1.1 (1)	1.1 (1)
Neutral	2.2 (2)	4.5 (4)
Confident	50.6 (45)	41.6 (37)
Very confident	46.1 (41)	52.8 (47)
Likelihood of future use		
Not at all likely		0 (0)
Not likely		1.1 (1)
Neutral		2.2 (2)
Likely		20.2 (18)
Very likely		76.4 (68)

Data include participants who vaccinated their children and used a strategy in phase 2 (*n* *=* 89). All variables in phase 1 refer to participants' perceived values. Likelihood of future use only assessed at phase 2.

IAM, Information Assessment Method.

Wilcoxon signed ranks test were run on the eligible sample of 102 participants and used to examine differences in responses to IAM and confidence variables across the 2 phases. Relevance significantly decreased in phase 2 compared with phase 1 (*Z* = −6.07, *P* < 0.001), as did understanding (*Z* = −2.52, *P* < 0.05), although these ratings remained positive. Confidence did not differ significantly across phases (*Z* = −0.65, *P* = 0.52), nor did anticipated and perceived benefits (χ^2^ (2) = 3.55, *P* = 0.17). There was a significant increase in positive cognitive impact across phases (*P* < 0.05, Fisher exact test).

#### 3.3.3. Predicted probability of actual strategy use and future use

A binary logistic regression was conducted to examine what variables in phase 1 predicted the actual use of a strategy in phase 2 (Table 5). The selected model included plans to use information and relevance in phase 1 as predictors. Plans to use information in phase 1 did not have a significant effect on strategy use in phase 2 (β = −1.85, *P* = 0.15); however, relevance in phase 1 did predict a greater probability of actual strategy use (β = 2.28, *P* < 0.01). Participants were 9.76 times more likely to report using a strategy if they find the information relevant than if they did not.

**Table 5:** Coefficients of the model predicting whether participants used a strategy.

Variable	*b*	95% confidence intervals for odds ratio		
		Lower	Odds	Upper
Included				
Constant	−2.69			
Plans to use information	−1.86	0.01	0.16	4.78
Relevance	2.28*	2.66	9.76	35.80

*R*^2^ = 0.00 (Hosmer and Lemeshow), 0.12 (Cox and Snell), 0.23 (Nagelkerke).

Model χ^2^ (2) = 13.44, *p* = 0.001.

* *p* < 0.01.

A second binary logistic regression was conducted to examine which variables predicted future strategy use (Table 6). The model included confidence in phase 2, which was found to significantly predict the probability of parents reporting plans to use a strategy again at a future vaccination (β = 1.44, *P* = 0.05). Participants were 4.23 times more likely to report plans for future strategy use if they were confident of using the information at the recent vaccination.

**Table 6:** Coefficients of the model predicting whether participants used a strategy and plans for future use.

Dependent variable	Independent variables	*b*	95% Confidence intervals for odds ratio		
			Lower	Odds	Upper
Strategy use	Included				
	Constant	−2.69			
	Plans to use information	−1.86	0.01	0.16	4.78
	Relevance	2.28*	2.66	9.76	35.80
Plans for future strategy use	Included				
	Constant	−2.60			
	Confidence	1.44*	1.00	4.23	17.90

Strategy use: *R*^2^ = 0.00 (Hosmer and Lemeshow), 0.12 (Cox and Snell), 0.23 (Nagelkerke).

Model χ^2^ (2) = 13.44, *p* = 0.001 *p < 0.01; Plans for future strategy use: *R*^2^ = 0.80 (Hosmer and Lemeshow), 0.04 (Cox and Snell), 0.16 (Nagelkerke). Model χ^2^ (1) = 3.70, *p* = 0.05.

* *p* = 0.05.

## 4. Discussion

The overall purpose of this study was to identify which variables relate to, and predict, planned, actual, and continued use of evidence-based pain management strategies for children's vaccination disseminated through an electronically available KT tool. These findings contribute new knowledge to the field of KT and vaccination pain management in children because no prior research has examined specific variables predicting parents' uptake of evidence-based practices presented in an electronically available KT tool for pediatric vaccination pain management.

The first hypothesis was supported as relevance of the information in phase 1 predicted a greater probability of parents' plans to use strategies at an upcoming vaccination. This suggests that situational relevance of the information to the parent is an essential factor to understanding what promotes use of a KT tool. Similar phenomena have been demonstrated in the e-health literature, where when health information is deemed relevant, parents are likely to feel reassured and confident in their choices and resulting behaviours from engagement with the acquired information.^30,48^ Thus, the current findings align with the literature suggesting that relevance of information is a key factor to promote parents' plans to use evidence-based practices in a KT tool, with confidence as a potential mechanism.

The second hypothesis was not met as plans to use strategies in phase 1 did not predict actual strategy use in phase 2. The disconnect between the planned and actual use of strategies is consistent with the behaviour change literature related to parent behaviour change and children's health. In a newborn pain management treatment randomized control trial, it was found that although nearly all parents expressed plans to breastfeed or use skin-to-skin care, parents rarely actually used these strategies in practice.^21^ One possible explanation for this disconnect is perceived behavioural control. Although perceived behavioural control is positively implicated in intentions to use information,^2,49^ it is argued to be less useful in predicting actual information use. This may be due to parents' overestimations of their control over a given situation, which could negatively relate to their ability to use information in the actual situation.^48^ In the present context, a parent may have planned to breastfeed during their infant's vaccination; however, the HCP administering the vaccination may have preferred the infant to be on the examination table. Another potential factor related to this disconnect may be decay of knowledge because participants may not have remembered the strategies between when they initially saw the tool and when the time came to actually use them. This is reflected in the finding that forgetting to use the tool at the time of vaccination was the second most common reason for not using the tool.

Contrary to plans in phase 1, relevance was predictive of strategy use. When information has personal relevance, the information is more likely to be attended to, processed, and recalled.^29,33,34^ In the broader immunization literature, when individuals have more knowledge about vaccines and pain management, the information is more likely to be used to inform decisions and subsequent action.^3,36,46,47^ Thus, perceived relevance potentially facilitated a more thorough level of cognitive processing, where individuals saw value in information use and thus were motivated to implement the KT tool. Taken together, relevance seems to be a pertinent variable to promote parents' use of pain management strategies during vaccination when the information is presented in a KT tool.

The third hypothesis was supported as confidence in strategy use at the time of vaccination predicted an increased probability of parents' plans to use the KT tool again in the future. This also aligns with the behaviour change literature, where individuals report greater confidence in their ability to implement a behaviour after previous positive experiences performing a behaviour.^22^ When parents report a higher degree of self-efficacy with regard to a health-related behaviour, it predicts intentions and confidence to engage in the behaviour, thus increasing the likelihood of behaviour uptake.^17,37,40^ Thus, the current findings align with the self-efficacy and health behaviour literature, with self-efficacy as a potential proxy promoting parents' plans to continue using the KT tool with their children at future vaccinations.

Our findings suggest that relevance of the KT tool, as well as confidence in using the information within the tool, is integral to parents' uptake of KT tools. Knowledge translation tool use, however, can be complicated by role confusion, including a lack of clarity between parents and HCPs on whose role it is to exchange and/or implement information on vaccination pain management.^41^ In this study, because information was directly conveyed to parents, the tool was relevant and directly addressed what parents could do. Therefore, having information tools that are relevant and tailored to parents' needs may promote the uptake of KT tools, given the clarity such tools can provide.

A novel contribution of this work is the importance of confidence in promoting KT uptake. It is well documented that there are significant challenges around understanding and implementing evidence.^39^ However, there is not much known about how confidence-building experiences in parents' use of evidence-based practices can influence KT uptake. Thus, the relationship between confidence and plans for future use of evidence-based practices within the KT tool is promising and could promote ongoing use of evidence-based practices in KT tools. A notable strength of this study is the 2-phase study design. Not only did this allow for understanding what led to plans to use the KT tool, but it also facilitated evaluation of whether those plans were brought to fruition. Furthermore, it allowed for the assessment of factors related to uptake, both at the vaccination appointment and for future appointments.

There are several limitations of this study including minimal diversity of the participants recruited. For example, expression, interpretation, and value of children's pain is known to differ cross-culturally and across parenting styles,^19^ and these differences could potentially influence how some parents would interact with such a tool. Relatedly, this study did not specifically look at uptake of specific strategies relative across child age. Future research should consider making a more directed effort in sampling from individuals from various ethnic, socioeconomic, and sex and gender backgrounds to account for diversity in experiences, while also considering the role of child age in utility of strategies. This is important to understand whether these factors play a role in the effectiveness of KT tools or even access to electronically available KT tools. It is also important to note the participant response rate of 41% at follow-up. This may have been related to lack of opportunity for a vaccination in the 6 months between the initial survey and follow-up. Future surveys could more clearly specify that all participants' responses are eligible, regardless of whether they had vaccinated after the first survey. In terms of contextual limitations, it is recognized that these results pertain primarily to vaccinations in traditional medical settings, and school-based vaccination pain management^42^ and needle pain management in other clinical settings^5^ requires additional consideration in terms of the applicability of these findings. Finally, this study used online, self-report surveys for data, which may have influenced sample representativeness. Future research should make an effort to ensure online surveys target a wide range of participants to ensure greater representativeness of the sample.

This study brings to light critical factors to consider when creating KT tools for parents' use with their children, including during children's vaccinations. These results highlight the importance of ensuring that the information communicated in KT tools are relevant to parents' needs and promote confidence to promote uptake of vaccination pain management strategies. The findings also highlight the importance of awareness of what information a given group requires, and it behooves researchers to work alongside knowledge users to ensure information in KT tools are, in fact, relevant and understandable. Children reap the ultimate benefits of the use of KT tools for vaccination pain management, as parent strategy use results in less painful and distressing vaccinations and encourages vaccine acceptance and adherence throughout the lifespan.

## Disclosures

The authors have no conflicts of interest to declare.

## Appendix A. Supplemental digital content

Supplemental digital content associated with this article can be found online at http://links.lww.com/PR9/A97.
